# Multipurpose, Integrated 2nd Generation Biorefineries

**DOI:** 10.1155/2016/4327575

**Published:** 2016-01-19

**Authors:** Alberto Scoma, Lorenzo Bertin, Maria A. M. Reis, Michael Kornaros, Marta Coma

**Affiliations:** ^1^Laboratory of Microbial Ecology and Technology (LabMET), University of Ghent, 9000 Ghent, Belgium; ^2^Department of Civil, Chemical, Environmental and Materials Engineering (DICAM), University of Bologna, 40131 Bologna, Italy; ^3^Department of Chemistry, UCBIO-Requimte, Faculty of Science & Technology, New University of Lisbon, 2829-516 Caparica, Portugal; ^4^Laboratory of Biochemical Engineering and Environmental Technology, Department of Chemical Engineering (DCE), University of Patras, 265 04 Patras, Greece; ^5^Centre for Sustainable Chemical Technologies (CSCT), University of Bath, Bath BA2 7AY, UK

The aim of the present special issue was to explore some recent advances in environmental biotechnology and chemistry with respect to the possibility of using bioresidues as renewable feedstock in the frame of 2nd generation biorefineries. As a matter of fact, pauperization of fossil resources is favouring the development of biorefinery platforms, where goods derive from biomass processing. Because of the ethical issues related to the use of food crops for fuel production, exploitation of organic residues present in agroindustrial, forestry, zootechnical, fishery, and municipal leftovers would enhance biorefinery competitiveness and social acceptance. This approach is referred to as “2nd generation biorefinery” and is still in its infancy, with the large majority of studies being conducted at laboratory scale and very few at pilot scale.

Among the major bioresidues/by-products generated by (agro)industries, lignocellulose is one of the cheapest and more abundant nonfood materials. It derives from plant biomass and it is made by 75% of polysaccharides, making carbohydrates an ideal basic platform for the generation of multiple products of societal and industrial interest. Lignin, another important compound of lignocellulosic-based biomass, may be also processed and/or extracted to increase the portfolio of such biorefineries. The present special issue majorly focuses on actual site lignocellulosic residues to produce either chemicals or fuels. Bioconversion of brewer spent grains (BSG) was described by R. Liguori, C. R. Soccol et al. using six* Lactobacillus* strains to generate lactic acid. Fermentation yields were compared with synthetic media also with respect to different pretreatments. Optimized cultivation reached as much as 22 g/L lactic acid. In P.-L. Tang et al. production of low molecular weight phenolic compounds was carried out using oil palm empty fruit bunch fiber (OPEFBF). While palm oil industry is rapidly expanding, an environmentally friendly solution to treat the lignocellulosic biowaste resulting from its processing is missing. P.-L. Tang et al. focused on the exploitation of lignin through enzymatic and chemical reactions following an alkaline pretreatment, which yielded a mixture of aromatic compounds such as hydroxybenzoic acid, vanillin, syringaldehyde,* p*-coumaric acid, and ferulic acid. V. Bátori et al. reported on the generation of multiple products using whole stillage as renewable feedstock, a by-product from ethanol production from dry-mill grains. Edible fungi such as* Neurospora intermedia* and* Aspergillus oryzae* were used for the integrated bioconversion of this organic residue into ethanol and protein-enriched fungal biomass. Economic implications following the upgrade of the present industrial process with the proposed scheme are discussed. This study has close connections with the one by R. Liguori, E. Ionata et al. who exploited the biotechnological potential of fungal activity to produce sugars. Here, cellulolytic and hemicellulolytic enzymes (a mixture of cellulases and xylanases) from* Pleurotus ostreatus* were used to optimize the saccharification yields of* Arundo donax* biomass. The influences of temperature, pH, and time as operational parameters were investigated in a 3^3^ factorial experimental design to find out the most critical ones. Significance of these experimental findings was described with respect to commercial enzymes. Finally, C. Nitsos et al. evaluated a number of methods to improve methane yields production by anaerobic digestion of three Mediterranean bioresidues. Pretreatment protocols applied hydrothermal, dilute acid, and steam explosion treatment to gain more energy from lignocellulosic residues such as olive tree pruning, grapevine pruning, and almond shells. Implementation of enzymatic hydrolysis was also tested for the lowest efficient treatments.

We believe that this collection of papers will be useful to people working in the area of green chemistry and biotechnology and will help to turn an environmental issue as waste disposal into a new opportunity for a sustainable biobased economy.

*Alberto Scoma* *Lorenzo Bertin* *Maria A. M. Reis* *Michael Kornaros* *Marta Coma*

